# How Our Gaze Reacts to Another Person’s Tears? Experimental Insights Into Eye Tracking Technology

**DOI:** 10.3389/fpsyg.2020.02134

**Published:** 2020-09-02

**Authors:** Alfonso Picó, Raul Espert, Marien Gadea

**Affiliations:** ^1^Department of Psychobiology, Faculty of Psychology, Universitat de València, Valencia, Spain; ^2^Centro de Investigación Biomédica en Red de Salud Mental (CIBERSAM)-Mental Health, Madrid, Spain

**Keywords:** crying, eye tracking, empathy, gaze, tears

## Abstract

Crying is an ubiquitous human behavior through which an emotion is expressed on the face together with visible tears and constitutes a slippery riddle for researchers. To provide an answer to the question “How our gaze reacts to another person’s tears?,” we made use of eye tracking technology to study a series of visual stimuli. By presenting an illustrative example through an experimental setting specifically designed to study the “tearing effect,” the present work aims to offer methodological insight on how to use eye-tracking technology to study non-verbal cues. A sample of 30 healthy young women with normal visual acuity performed a within-subjects task in which they evaluated images of real faces with and without tears while their eye movements were tracked. Tears were found to be a magnet for visual attention in the task of facial attribution, facilitating a greater perception of emotional intensity. Moreover, the inspection pattern changed qualitatively and quantitatively, with our participants becoming fully focused on the tears when they were visible. The mere presence of a single tear running down a cheek was associated with an increased emotional inference and greater perception of sincerity. Using normalized and validated tools (Reading the Eyes in the Mind Test and the SALAMANCA screening test for personality disorders), we measured the influence of certain characteristics of the participants on their performance of the experimental task. On the one hand, a higher level of cognitive empathy helped to classify tearful faces with higher emotional intensity and tearless faces with less emotional intensity. On the other hand, we observed that less sincerity was attributed to the tearful faces as the SALAMANCA test scores rose in clusters A (strange and extravagant) and B (immature and emotionally unstable) of our sample. The present findings highlight the advantages of using eye tracking technology to study non-verbal cues and draw attention to methodological issues that should be taken into account. Further exploration of the relationship between empathy and tear perception could be a fruitful avenue of future research using eye tracking.

## Introduction

In humans, emotions are automatically transmitted through visual cues, including non-verbal behaviors such as facial expressions and body language (Kret, 2015). Among all the signals by which emotions can be expressed, visible tears – and more specifically the shedding of tears in response to an emotional state, as opposed to those in response to pain or a physical irritation of the eye – are one of the most ubiquitous displays of human emotional. Recently, the socioemotional impact of visible tears on others’ perceptions and judgments is receiving growing and deserved attention as a field of empirical study (for an up-to-date non-systematic metanalysis on emotional crying, see Zickfeld et al., 2020). However, no previously published eye tracking studies have employed objective measures than self-reporting to throw light on reactions to emotional crying. We decided to apply eye tracking technology and a carefully selected series of stimuli to answer the question “How our gaze reacts to another person’s tears?” The eye tracking technique has a long history (Yarbus, 1967) and allows gaze measures to be assessed with respect to the so-called “tearing effect.” With the present work, we also set out to offer methodological insight and advice on how to use eye tracking technology to study non-verbal cues by providing an illustrative example of an experimental setting specifically designed to study the “tearing effect.”

### Gaze Measures, Before and Now

**FIGURE 1:**
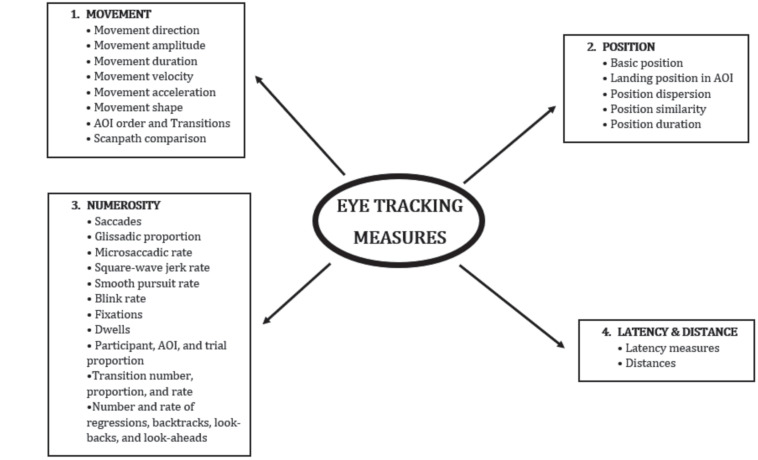
Fundamental classification of modern eye tracking measures. This chart summarizes the taxonomy proposed by Holmqvist and Andersson (2017) for the basic classification of 120 eye tracking measurements reviewed in the literature. The number of different measurements is increasing over time due to the combination of some of the existing ones, as well as the creative use of technology that provides the opportunity to find new and interesting metrics.

The measurement of oculomotor variables in cognitive science dates back more than 100 years (Dodge and Cline, 1901; Dodge, 1903) and constitutes a non-invasive method for evaluating a wide variety of processes, from emotional recognition to social information processing (Xiao et al., 2015). Gazing is unique among non-verbal behaviors in that the eye is a sensory organ for gathering information and, at the same time, performs the function of a signal to others (Harrigan et al., 2005). However, of the more than 1,700 articles on gaze published since 1982 and included in the review by Harrigan et al. (2005), only 13% investigated non-verbal behaviors. More recently, in a short review on the research conducted over the past 5 years, the keywords gaze and non-verbal behavior in Google Scholar, MEDLINE, Pubmed, and Scopus yielded 17,700 results. Unfortunately, this current emphasis has not always been accompanied by clear explanations about the best methodology for conducting such studies. In particular, there are very few descriptions of the methodology used to codify gaze (with the exception of some classic works, such as those by Exline and Fehr (1982) and Fehr and Exline (1987). In non-verbal research, gaze measures have traditionally been divided into (1) frequency, (2) total duration of the gaze, (3) proportion of time looking at, (4) average duration of individual glances, (5) standard deviation of glances, and (6) mutual gaze (the most investigated) (Argyle and Ingham, 1972). Other authors have determined different forms of eye movements based on their duration (Kirkland and Lewis, 1976). These traditional categorical classifications have largely been superseded by a quantitative approach that makes use of detailed records of eye movements through so-called eye tracking devices, which measure nearly 120 different metrics corresponding to basic properties of movement, position, numerosity, latency, and distance of the gaze (see Figure 1 for an overview). Eye tracking technology has been widely used to analyze stimuli of different emotional valence in order to throw light on differences in visual behavior (Tavakoli et al., 2015; Rubo and Gamer, 2018) and how subtle differences can lead to major changes in gaze behavior. In addition, the quantitative evaluation of facial emotional expressions by eye tracking technology has provided useful insight into child and adolescent psychiatry (Rommelse et al., 2008), neurodegenerative diseases (Bek et al., 2020), mood disorders (Peckham et al., 2016; Hunter et al., 2020), and other behavioral disorders (Martin-Key et al., 2017). In this way, it is the perfect tool for our interests and presents itself as the logical next step in the investigation of emotional crying (Krivan and Thomas, 2020). Nonetheless, there are some methodological flaws that are repeated over and over again in studies employing eye tracking methodology. Some of them can be rectified using statistical techniques that take into account the special characteristics of the analyzed data (e.g., the vast majority of eye tracking metrics do not follow a Gaussian distribution). Others can be overcome by an appropriate experimental design (e.g., eye tracking metrics are idiosyncratic for most participants and stable across trials, so comparisons between groups of subjects can be problematic) or by controlling variables like gender or a misuse of the signal-to-noise ratio that could make any spurious result statistically significant in a sufficiently large sample. These and other problems are perfectly solvable if one understands the methodology of eye tracking, which will be detailed later, but they become especially problematic for researchers of non-verbal communication, as became evident when we performed a review of the literature.

### Tears and Emotional Crying

Theoretical positions rooted within an evolutionary framework have suggested that tears act as biological signals, which serve to express a request for help (Roes, 1989; Kottler, 1996; Walter, 2008). Furthermore, the literature points to the functional role of emotional crying as a form of communication, for example, of the need for attention and support (Hendriks and Vingerhoets, 2006) and the need to be perceived as warmer and friendlier (van de Ven et al., 2016; Vingerhoets et al., 2016; Zickfeld et al., 2018) or more honest (Zeifman and Brown, 2011). Provine et al. (2009) claimed that emotional tears can improve emotional recognition, at least with regards to the sad feelings of the crier. These authors, and others (Provine et al., 2009; Zeifman and Brown, 2011), have proposed the “tearing effect” as a sign of improved emotional perception and processing of facial expressions in the presence of tears, which eliminates the potential emotional ambiguity toward the observed face. Importantly, it has been suggested that tears exert their intended influence provided that they are perceived as “natural;” that is, if tears are depicted running upward instead of downward from the eye (in an unnatural direction), they lose their emotional impact (Provine, 2014, 2017). This indicates that tears are a special stimulus (i.e., emotional signal) that have priority over others (Vuilleumier et al., 2002; Killgore and Yurgelun-Todd, 2004). In summary, the literature as a whole bears testimony to the fact that the perception of emotional tears, even when operating at a preattentive level (Balsters et al., 2013; Lockwood et al., 2013), is capable of inducing important behavioral changes in the observer.

### Objectives and Hypothesis

In light of the above scenario, we were interested in measuring the changing gaze behavior of observers with an objective methodology (eye tracking) and in investigating some of the putative functional roles of tears. Moreover, based on our own experience, we felt it would be useful to offer methodological advice on the use of eye tracking technology for the study of non-verbal cues beyond muscle activation in facial expressions (Kret, 2015). We argue in favor of a particular type of experimental design over others and for the selection of an appropriate sample size and eye tracking measures. Thus, we designed a study to explore some basic eye tracking measures during the observation of calm crying faces (i.e., duration of gazing and number of fixations within an area of interest – henceforth AOI – where the tear appears). We hypothesized that tearful faces would receive longer gaze time inside the AOI and that the AOI of tearful faces would receive more dwells and a greater number of fixations. With regards to the functional roles of crying, we expected that the presence of tears would facilitate the perception of the emotional intensity of the subjects’ faces (related to the tearing effect), lead participants to perceive the subjects to be more sincere (related to the perception of more honesty), and elicit more sympathy from our participants toward the subjects (related to the proposed function of tears in communicating the need for help), considering sympathy as an affective experience with a prosocial motivation toward others (to help or relieve the suffering) (Walter, 2012).

An additional aim of this study was to consider the influence of factors inherent to the observer’s perceptual processing of tears. The interesting review of Vingerhoets and Bylsma (2016) suggested that the study of crying was the “gateway” to achieve a better insight into important developmental processes like empathy and personality disorders. We hypothesized that people scoring high in cognitive empathy would be more prone to experiment the “tearing effect.” Regarding personality features and crying, individuals with high levels of neuroticism cry relatively more (Peter et al., 2001), whereas dismissively attached people tend to cry less than others (Laan et al., 2012). Moreover, the crying of patients with borderline or narcissistic personality disorders can be perceived as manipulative and annoying by therapists in clinical settings (Alexander, 2003). Given such observations about crying with respect to personality disorders, we wondered whether the observation of other people’s crying would also reveal a relation to personality disorders when measured in a non-clinical sample.

## Materials and Methods

### Participants

Taking into account the experimental design, and on the basis of data from a previous pilot study (Picó et al., 2018), we performed a power analysis to justify the detection of medium effect sizes with a probability of 0.8 for a paired sample test. Subsequently, this power analysis was used to select a convenient sample size of 27 participants, but it was not employed in the correlational analyses, which occupy a secondary role in the present work. The use of power analyses that justify the sample size is essential to avoid problems of signal–noise discrimination that could cause us to incur in type I errors. To perform the analysis, we used the “pwr” package (Champely, 2018) from R software. Thirty undergraduate women aged 18–27 years (*M* = 22.23, *SD* = 2.39) were recruited from the Nursing degree at the University of Valencia (Spain) and were given a 16 GB USB memory stick as a reward for their participation in the experiment. Selection criteria included a near-perfect vision (no glasses or contact lenses), no reported history of psychiatric disorders, and no chronic pharmacological treatment. Two individuals were excluded from the eye tracking data collection due to technical issues. All the participants were treated in accordance with the “Ethical Principles of Psychologists and Code of Conduct” and the precepts of our university’s Ethics Committee, and all signed an informed consent form.

### Materials

#### Visual Stimuli

We used a set of four photographs of neutral faces of adult persons – two women and two men – kindly provided by the photographer Marco Anelli. These photographs had been used in previous studies (van de Ven et al., 2016; Vingerhoets et al., 2016; Zickfeld et al., 2018; Stadel et al., 2019; Picó et al., 2020). The photographs were taken in the precise moment when the subject was engaged in calm crying – the particular distinction of which is the presence of visible tears with little marked emotional expression – in a spontaneous way (see details of the photographs in Picó et al., 2020). The images were manipulated to digitally remove the visible tears so that the experiment was carried out with a total of eight images: four with tears and four without, representing both genders in each case. In addition, the facial expression in each photograph was accompanied by a text consisting of an explicit affirmation (e.g., “I am not cheating on my boyfriend!”) as if the phrase was being pronounced by the subject. We wrote four vignettes of text, one for each of the four subjects depicted in the photographs, and each text was paired with the two versions of the photo of the same person, once with the photo showing tears and once with the photo without tears. The order of the four photos and vignettes was completely counterbalanced. Prior to the experiment, we carried out a practice trial (not analyzed) in which the participants looked at the pictures of two women with neutral facial expressions (i.e., AF05NES and AF23NES) extracted from the Karolinska Directed Emotional Faces (Lundqvist et al., 1998), with their corresponding vignettes. The rationale for using images that depict calm crying expressions lies in the assumption that, if the effect of emotional crying is mainly due to the presence of tears, it will be detectable even in faces with little emotional display (Vingerhoets, 2013).

#### Eye Tracker Device

The device that we used to measure the visual variables related to attentional factors was a 150 Hz GP3 HD UX eye tracker system (Gazepoint systems, Toronto, Canada) connected to a PC with a 19″ LED Benq GL950 Senseye monitor. This eye tracker model has a wide lens, allowing relatively large head movements to be monitored during experimental tracking (∼35 cm in horizontal movement and 22 in vertical movement), without the need to restrain participants; even so, our participants were instructed to remain as still as possible, with their backs straight, up against the back of the chair. We processed the experimental data with Gazepoint Analysis UX software (Gazepoint Systems, Toronto, Canada). The most basic eye tracking data – from which the rest of the metrics can be calculated – are X- and Y-coordinates of the fixation point of gaze, measured as a fraction of the screen size at specific times (in our case, every 1/150 s). The point of gaze (POG) used is the average of the left eye and right eye POG if both are available; if not, the value of either the left or right eye is used, depending on which one is valid.

#### Questionnaires

The “Reading the Mind in the Eyes” test, also known as RMET (Warrier et al., 2017a), was administered as a brief social cognition test to measure cognitive empathy. Cognitive empathy is a construct closely related to Theory of Mind (ToM). Specifically, ToM refers to the ability to represent and understand, in general, the mental states of others. Cognitive empathy refers to the ability to understand and mentalize about the feelings of others, considering feelings to be a mental state among others, without necessarily implying that the empathizer is in an affective state himself (Walter, 2012). We chose cognitive empathy because, according to Warrier et al. (2017b), enhanced cognitive empathy results in a higher ability to recognize another person’s mental states. In this test, a series of 36 photographs depict eye regions from different models who express a range of emotional states. Four words are presented at the same time, surrounding the photo, and each word refers to a unique mental state. Participants are asked to choose which one of the four words better suits what the person in the photograph is feeling.

The Personality Disorders Screening Test SALAMANCA questionnaire (Pérez-Urdániz et al., 2011) was administered as a brief screening tool for evaluating personality in our sample of participants. This instrument evaluates the presence of 11 personality disorders drawn from the Diagnostic and Statistical Manual of Mental Disorders (DSM) (paranoid, schizoid, schizotypal, histrionic, antisocial, narcissistic, and dependent) and the International Classification of Diseases (ICD) (emotionally unstable personality disorder-impulsive type, emotionally unstable personality disorder-borderline type, also known as limit, anankastic, and anxious). These 11 disorders are classified in three groups: Type A, strange and extravagant (paranoid, schizoid, and schizotypal); Type B, immature (histrionic, antisocial, narcissist, and both subtypes of emotional unstable disorders: impulsive and limit); and type C, avoiding (anankastic, dependent, and anxious). The SALAMANCA tool consists of a total of 22 questions; each personality trait is evaluated through two questions using a 4-point Likert scale (*false* = 0 points; *sometimes true* = 1 point; *usually true* = 2 points; *always true* = 3 points). The cutoff score is established at 3 points for every trait. This questionnaire has been validated and correlated with the Interpersonal Personality Disorder Examination and is considered an adequate test of screening, with a sensitivity of 100% and a specificity of 76.3% (Caldero-Alonso, 2009). It is important to note that this questionnaire is not intended as a diagnostic tool but rather for screening tendencies to suffering personality disorders (vulnerabilities), which should be confirmed by a psychiatrist in every case. It is a self-assessment questionnaire (< 10 min) that is easily interpreted.

### Procedure

Before each participant performed the task, the eye tracking system was calibrated according to a standard protocol with nine calibration positions on the screen in order to be sufficiently personalized. The monitor was positioned 67 cm from the eyes of the participant (equally for the entire sample). Following calibration, participants carried out the task of viewing the photos of the faces with and without tears. Before each stimulus, participants were told they would be presented on the computer screen with a statement (a text vignette, for 15 s) and that they would then see the face of the person who had said the message in the text (the photograph, for 2 s). The gazing of the participants was eye tracked only while the photos were presented on the screen. Note that the photographs appeared on the screen for a very short time; this is an important methodological issue with respect to analysis of the data provided by eye tracking measures, known as “dependence between successive measurements” (Tatler and Vincent, 2008), which is rarely taken into account. The longer the stimuli is displayed on the screen, the greater are the potential bindings of the data, and classical statistical tests do not provide reliable results in this particular circumstance. One of the easiest ways to simplify the situation in eye tracking systems of < 250 Hz is to ensure that the stimulus is available on the screen for a short time (for example, for 2 s, as in the present study). Of course, this strategy is not free of problems, and the duration of the stimulus depends on the expected size of the effect to be detected and the nature of the study (Andersson et al., 2010).

**FIGURE 2:**
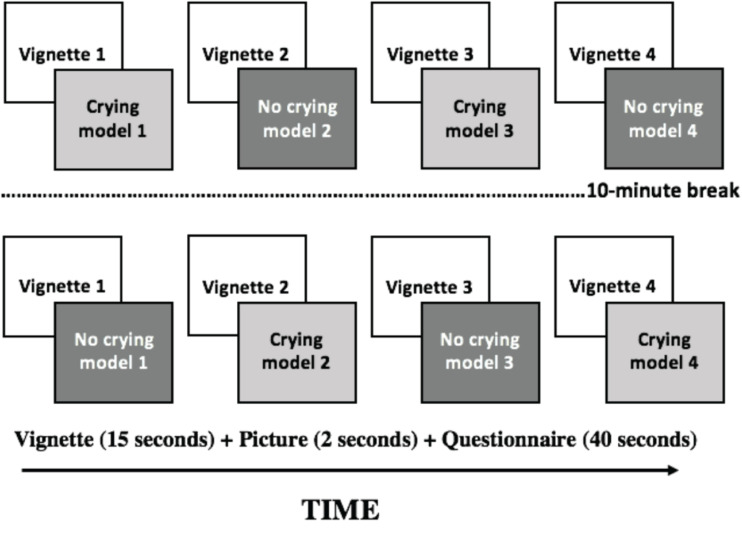
Schematic representation of the experimental procedure for participants. Each participant was presented with the text/vignettes and the faces in a different order to avoid carryover effects. Each vignette was tailored and attached to each model. In this way, visible tears were the only difference between the two conditions.

Our participants were instructed to read the text and to observe the corresponding face carefully, as afterward, they would be asked to complete a questionnaire about what they had seen. In this way, immediately after the visual task, participants were given 40 s to respond to a number of questions about the stimuli on a sheet of paper (see a schematic representation of the experiment design in Figure 2; see details of the questionnaire in the section below). As shown in Figure 2, participants were presented with a first round of four text vignettes plus faces and completed the corresponding questionnaire after seeing each face. Next, the participants were told that they should relax their eyes for 10 min by sitting quietly in a comfortable chair, with their eyes closed and covered with an eye mask. Following this 10-min break, they repeated the task with the same pictures but with/without visible tears (note that the order of presentation was counterbalanced). At this point, we would like to argue in favor of within-subject experimental designs (or repeated measures designs) when using the eye tracking device due to the high variance in this measure among participants (Andrews and Coppola, 1999; Rayner, 2009; Johansson et al., 2012). Between-subject designs require a large number of participants in order to reach an acceptable power to perform a parametric evaluation of statistical differences. The main disadvantage of within-subjects designs is that the order in which the stimuli are presented can affect the validity of the causal inference process (Duchowski, 2017), and the effects of learning and fatigue are further disadvantages. However, these drawbacks can be mitigated perfectly by counterbalancing the presentation of stimuli and by employing short tasks to be carried out in less time, as we did in the present experimental setting. Finally, once we had collected all the data regarding the visual stimuli, participants were asked to complete the RMET and SALAMANCA questionnaires, after which they were given their gift and thanked for their participation.

### Measures and Dependent Variables

We recorded three types of measures: measures related to the visual stimuli and the subjective reaction of the participants to them; gaze measures related to the visual stimuli and obtained by means of the eye-tracker system; and, finally, empathy and personality measures of the sample using the RMET and SALAMANCA questionnaires, respectively. Regarding the subjective measures related to the visual stimuli, each photograph of a face, with its attached text, was followed by a questionnaire, which included the following items: (1) the degree of intensity of emotionality the face seemed to show, (2) the perceived sincerity in the observed face with respect to the corresponding statement made by that person (the paired text), and (3) whether the observed face evoked sympathy (or not) in the participant. All questions were assessed on a 6-point Likert scale, where 0 indicated the complete absence of intensity, sincerity, or sympathy and 5 the highest degree of each. Regarding the gaze measures obtained through the eye tracking device, we hand-drew an area of interest (AOI) in the form of a rectangle framing both eyes and widened below the right eye to the right cheek (where tears were visualized on the crying faces), in accordance with Goldberg and Helfman (2010) advice that AOIs should be defined only on objects of interest. We measured the following dependent variables: (1) duration of the gaze inside the AOI in milliseconds, (2) fixations on the AOI (a fixation is defined as maintaining the gaze in a square of 1-degree amplitude for at least 100 ms), (3) revisits or dwells (i.e., looking at the AOI more than once), (4) number of fixations on the global stimuli (i.e., inside and outside the AOI), and (5) mean duration of said global fixations. These eye tracking metrics are available in the vast majority of current software, and we chose them to facilitate future replication of our results by other researchers. All the metrics can be calculated from eye tracking records between 60 and 2,000 Hz, so they are not restrictive with respect to the equipment that can be used.

### Data Analysis

Data management and analysis were performed using the statistical software R version 3.6.0 (2019), R package *WRS2* (Mair and Wilcox, 2020), and *psych* (Revelle, 2018). Before applying parametric methods, we performed Shapiro–Wilks tests to check the normal distribution of the data. Since some of our variables were skewed (as expected), we selected a robust *t*-test for paired samples with bootstrapping (*n* = 1,000) to analyze differences between the tear and no tear conditions in terms of intensity of the gaze inside the AOI, fixations on the AOI, revisits of the AOI, global fixations (number), and global fixations (time). To ensure that the total duration of fixations (inside and outside the AOI) did not influence the results, we performed an analysis of covariance (ANCOVA) of the total duration time of fixations as a covariate, and the results did not reveal a significant effect of the total duration on any measure. The explanatory measure of effect size ε reported in this analysis is a robust version (Wilcox and Tian, 2011; Mair and Wilcox, 2020), which does not require equal variances and can be generalized to multiple group settings. As a reference, ε = 0.10, 0.30, and 0.50 correspond to small, medium, and large effect sizes. In addition, Pearson’s product–moment correlations were used to test whether personality traits and/or level of empathy of the participants were related to the experimental results. Results were significant at the *p* < 0.05 level, and *p*-values were corrected with Bonferroni’s method for multiple comparisons. The use of robust parametric statistics (as in our case) that take into account the transgression to some of the fundamental requirements of the classical models (i.e., Gaussian distribution, independency, and homoscedasticity), or relevant transformations in dependent variables, is necessary when working with eye tracking data. We recommend a balance between the most appropriate techniques and those that are simple to interpret.

## Results

### Intensity of Emotion, Perceived Sincerity, and Evoked Sympathy

On average, crying faces (trimmed *M*_tearful_ = 3.70) elicited a significantly higher mean perception of emotional intensity [*t*(17) = 6.48, *p* = 0.000, ε = 0.75] than the faces without visible tears (*trimmedM*_tearless_ = 2.85). The crying faces were also perceived to be significantly more sincere than the same faces without tears [*t*(17) = 3.02, *p* < 0.01, ε = 0.34] with trimmed *M*_tearful_ = 3.68 and *M*_tearless_ = 3.34, respectively. The crying faces (trimmed *M*_tearful_ = 3.21) evoked a higher mean sympathy than the tearless faces (trimmed *M*_tearless_ = 3.09), although this value did not reach statistical significance [*t*(17) = 0.58, *p* = ns]. A summary of these results can be found in Table 1.

**TABLE 1:** Descriptive statistics with robust paired *t*-test results.

	TEARFUL					TEARLESS					
	*n*	Mean	*SD*	Median	Trimmed	*n*	Mean	*SD*	Median	Trimmed	Yuen’s t
Intensity	30	3.66	0.51	3.62	3.70	30	2.83	0.65	2.75	2.85	6.48***
Sincerity	30	3.70	0.61	3.75	3.68	30	3.33	0.72	3.50	3.34	3.02**
Sympathy	30	3.17	0.69	3.25	3.21	30	3.01	1.10	3.00	3.09	0.58
Duration AOI	28	751.21	515.76	795.00	725.08	29	305.72	233.52	256.00	294.48	3.38**
Fixations AOI	28	1.89	0.92	2.00	1.92	29	1.28	0.92	1.00	1.24	1.22*
Revisits AOI	28	0.75	0.65	1.00	0.71	29	0.76	0.83	1.00	0.68	0.56
Fixations^a^	29	9.97	0.87	10.00	10.00	30	9.43	1.04	9.00	9.46	4.40***
Fixations (time)^b^	28	19.68	1.97	19.47	19.53	30	20.11	2.62	19.93	20.02	−0.77

*p < 0.05, **p < 0.01, ***p < 0.001. Trimmed means were calculated with a trim level of 0.2. n reflects the final number of cases used in the analysis. ^*a*^Number of fixations inside and outside the AOI. ^*b*^Duration of fixation inside and outside the AOI. Crying faces were also perceived to be significantly more sincere, and with more emotional intensity. Participants spent significantly more time gazing faces with tears. Moreover, the number of fixations inside the area of interest and overall fixations were higher when inspecting crying faces.

### The Effect of Visible Tears on Eye Tracking Measures

With regard to eye tracking data, participants spent significantly more time gazing (duration measured in milliseconds) inside the AOI of crying (trimmed *M*_tearful_ = 725.08) vs. non-crying faces (trimmed *M*_tearless_ = 294.48), *t*(17) = 3.38, *p* = 0.003, with an explanatory effect size of εε = 0.66. The number of fixations inside the AOI was also significantly higher with respect to the crying faces (trimmed *M*_tearful_ = 1.92 and *M*_tearless_ = 1.24) [*t*(17) = 1.22, *p* = 0.015], with an effect size of 0.60 and a median difference of one fixation. The number of revisits was not statistically significant *t*(17) = 0.56, *p* = ns, with a trimmed mean difference of 0.11 and an ε = 0.1. With regards to gaze fixations and duration of the fixations on the whole stimuli (AOI plus outside the AOI), participants engaged in significantly more fixations [*t*(18) = 4.40, *p* < 0.000, ε = 0.59] on the crying faces (trimmed *M*_tearful_ = 10 and *M*_tearless_ = 9.43), with no significant differences in the duration of such fixations [*t*(17) = −0.77, *p* = ns, ε = 0.14] between the two faces. These results are summarized in Table 1.

### Influence of Empathy and Personality Traits of the Sample

Regarding the scores of the RMET test for measuring cognitive empathy, we observed that the higher the RMET score was, the more emotionally intense the crying face was perceived to be (*r* = 0.48, *p* < 0.01, see the correlations regarding tearful faces in Table 2). Interestingly, we also observed that, as the RMET score increased, the non-crying face was perceived to be less intense (*r* = −0.44, *p* < 0.01, see correlations regarding tearless faces in Table 3). However, no correlations were observed between RMET levels and eye tracking measures for any of the two conditions (see Tables 2, 3).

**TABLE 2:** Correlations in the tearful condition.

	Intensity	Sincerity	Sympathy	Fixations	Duration of fixations	Duration (AOI)	Fixations (AOI)	Revisits (AOI)
RMET	0.48**	–0.03	–0.01	0.50	0.23	–0.12	–0.07	–0.26
Paranoid^a^	0.42*	–0.16	0.13	0.47	0.49	–0.31	–0.40	–0.44
Schizoid^a^	–0.15	−0.50**	–0.08	0.01	0.05	0.21	0.08	0.19
Schizotypal^a^	0.09	−0..56**	–0.17	0.17	0.55	–0.21	–0.35	–0.30
Histrionic^b^	–0.02	–0.14	0.08	0.12	0.40	–0.08	–0.34	–0.14
Antisocial^b^	0.06	−0.59**	–0.20	–0.03	0.70**	–0.21	–0.28	–0.22
Narcissistic^b^	−0.36*	−0.54**	–0.33	–0.42	0.52	–0.44	–0.56	–0.50
Impulsive^b^	–0.22	−0.47**	–0.13	–0.07	0.36	0.18	–0.11	0.18
Limit^b^	0.11	−0.39*	–0.04	0.17	0.15	0.11	–0.33	0.05
Anankastic^c^	0.17	–0.05	0.12	–0.05	0.22	0.02	–0.37	–0.13
Dependent^c^	–0.31	–0.27	0.05	0.01	0.24	0.00	–0.14	0.08
Anxious^c^	–0.06	–0.19	0.05	0.36	0.27	–0.08	–0.31	–0.50

*p < 0.05, **p < 0.01. ^*a*^Type A personalities in the SALAMANCA screening test (strange and extravagant). ^*b*^Type B personalities in the SALAMANCA screening test (immature and subtypes of emotional unstable disorders). ^*c*^Type C personalities in the SALAMANCA screening test (avoiding). The higher the RMET score was, the more emotionally intense the crying face was perceived to be. Low narcissism and higher paranoid ideation were related to the perception of a more intense emotionality in the faces with visible tears, meanwhile a higher schizoid or schizotypal score was negatively associated with the perception of sincerity. The antisocial tendencies along with high vulnerability to narcissism were related to low attributed sincerity. Moreover, emotional instability disorders correlated negatively with perceived sincerity.

**TABLE 3:** Correlations in the tearless condition.

	Intensity	Sincerity	Sympathy	Fixations	Duration of fixations	Duration (AOI)	Fixations (AOI)	Revisits (AOI)
RMET	−0.44*	–0.32	–0.27	0.23	–0.17	–0.40	–0.37	–0.31
Paranoid^a^	–0.02	–0.23	–0.11	–0.02	0.14	–0.01	0.00	0.05
Schizoid^a^	0.24	–0.26	–0.13	0.14	–0.10	0.25	0.26	0.41
Schizotypal^a^	0.04	–0.31	–0.27	0.00	0.14	0.28	0.30	0.53
Histrionic^b^	0.00	–0.07	–0.01	–0.50	0.59	–0.17	0.14	0.33
Antisocial^b^	0.09	–0.20	–0.28	0.01	0.13	0.29	0.42	0.61
Narcissistic^b^	0.23	–0.12	–0.15	–0.38	0.35	0.27	0.03	0.05
Impulsive^b^	–0.17	–0.27	–0.11	–0.45	0.46	–0.27	–0.09	0.13
Limit^b^	0.04	–0.31	–0.15	–0.20	0.28	–0.10	0.13	0.34
Anankastic^c^	0.27	0.05	0.08	–0.38	0.46	0.12	0.40	0.54
Dependent^c^	0.17	–0.21	0.01	–0.44	0.34	–0.14	–0.06	0.13
Anxious^c^	–0.08	–0.23	–0.09	–0.39	0.51	–0.13	0.12	0.35

*p < 0.05. ^*a*^Type A personalities in the SALAMANCA screening test (strange and extravagant). ^*b*^Type B personalities in the SALAMANCA screening test (immature and subtypes of emotional unstable disorders). ^*c*^Type C personalities in the SALAMANCA screening test (avoiding). As can be seen, as the RMET score increased, the non-crying face was perceived to be less intense.

With regards to personality measured with the SALAMANCA screening test for vulnerability to personality disorders, the most relevant result was that correlations were significant when the participants were presented with the tearful faces and not when they were presented with the non-crying faces. The emotional intensity of the faces was inversely and significantly correlated to the narcissistic score (*r* = −0.36, *p* < 0.05) and positively and significantly correlated to the paranoid score (*r* = 0.42, *p* < 0.05); thus, low narcissism and higher paranoid ideation were related to the perception of a more intense emotionality in the faces with visible tears. In the case of sincerity, a higher vulnerability to personality disorders was generally related with a lower sincerity attributed to the tearful face. Specifically, a higher vulnerability to schizoid or schizotypal disorders was negatively associated with the perception of sincerity (*r*’s = −0.50 and −0.56 with *p*’s < 0.01). A personality with antisocial tendencies was inversely related to attributed sincerity (*r* = 0.59, *p* < 0.01). High vulnerability to narcissism was also related to low attributed sincerity (*r* = −0.54, *p* < 0.01). Lastly, vulnerability to emotional instability disorders (i.e., limit and impulsive) correlated negatively with perceived sincerity (*r*’s = −0.39 and −0.47, with *p* < 0.05 and *p* < 0.01, respectively). It should be stressed that all the above results refer to the tearful faces and that we did not find any significant correlation among these personality measures and the attributions of emotional intensity, sincerity, or sympathy elicited by the faces without visible tears. Finally, these personality measures were not closely related to the gaze measures obtained with the eye tracker. Once again, we found no relation when judging the non-crying faces, but when faces with visible tears were viewed, we observed that the antisocial personality score rose with the duration of visual inspection outside the AOI (*r* = 0.70, *p* < 0.05). Tables 2, 3 summarise the correlational results.

### Heatmaps and Fixations: A Qualitative Inspection (Figure 3)

**FIGURE 3:**
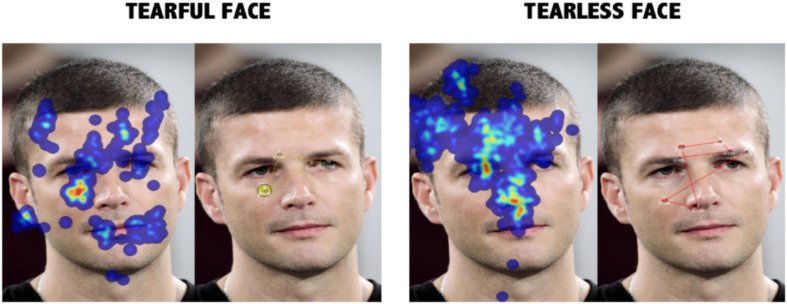
Overall heatmap and fixations map in model 4. The overall effects represent a clear change in the pattern of gaze in model 4. Not only was the time within the zone delimited by eyes and right cheek superior in the tearful condition, but also a qualitative change was also observed.

As an example, Figure 3 is a graphical representation of the average visual behavior observed when a face (i.e., model 4) was explored. We can observe the triangular geometric pattern that runs from one eye to the next and then down to the mouth and then back to the first eye (Iskra and Gabrijelcic, 2016), with extents of preference in the eyes–mouth continuum (Rogers et al., 2018) when tearless faces were judged and with brief fixation times and spreading points over the face. As the figure shows, the presence of tears alters the visual inspection pattern, breaking the triangle of fixations concentrating them inside the AOI, as if tears were powerful visual attention magnets.

## Discussion

The main objectives of this study were to evaluate some of the suggested functional roles of tears and to explore the modification of gaze behavior when subjects are presented with faces with visible tears. Earlier research carried out in our laboratory showed how visible tears are capable of altering inferences regarding emotional intensity and sincerity perceived in human faces (Picó et al., 2020). In the present experiment, we replicated some of the results of our previous study with regards to faces engaged in calm crying. The participants in the present sample perceived the emotional expression of the faces to be more intense and judged it to be more sincere. Our results are also in line with previous evidence that tearful faces can facilitate the perception of emotional expression (Vingerhoets, 2013; Vingerhoets et al., 2016; Gračanin et al., 2017). Weeping is a genuine way to show emotion and is usually associated with sadness (Klineberg, 1940; Van de Ven et al., 2017) but can also occur in happy situations (Vingerhoets and Cornelius, 2001). The present study shows how tears convey a message without the explicit need to identify the specific emotion that caused them. We believe this finding is especially interesting given that we have evaluated tears in calm crying faces. As Ito et al. (2019) recently pointed out (2019), visible tears seem to constitute a context in themselves that facilitates emotional inference, even in the absence of any other emotional clue. In addition, our participants judged the phrases associated with the crying faces as being more sincere. In this way, calm crying faces exerted an influence on sincerity as a state, in accordance with Zeifman and Brown (2011), who reported that the presence of visible tears increased the perception of honesty (sincerity as a trait) in subjects, and with Regan and Baker (1998), who showed that the testimonies of children who had been victims of sexual assault were perceived as more credible if they cried. According to Van Kleef (2008)’s theory of emotion as social information (2009), people use the perceived emotions of others to clarify ambiguous social situations. It is possible that an emotional sign such as visible tears makes it easier to label a specific social situation, and this might help to generate a greater sense of sincerity in the communication. If this were the case, visible tears would represent a non-verbal clue indicating sincerity, a quality that is indispensable for a fruitful collaboration in an ultrasocial species such as ours (Tomasello, 2014). Such a clue could be used by dishonest individuals in order to take advantage of their peers, and indeed, crying is also seen as one of the most conventional tactics of emotional manipulation (Buss, 1987). As for the sympathy aroused in our participants by the tearful faces, though it was greater than that provoked by the tearless versions, it did not reach statistical significance.

In contrast, the results of the eye tracking task revealed profound changes in gazing behavior provoked by crying faces. The presence of visible tears led to a greater visual inspection of the eyes and right cheek, where the most pronounced visible tear was located. The participants not only spent more time looking at this AOI in the crying faces, but they also engaged in more fixations there (i.e., they maintained their gaze on a fixed point inside the AOI more times when this area contained tears). As Loftus (1972) demonstrated, scene recognition can be expressed as a positive function of the number of fixations, and in the present study, we detected a significantly greater number of fixations in the tearful condition. The literature demonstrates that the enrichment of general stimuli leads to a greater number of fixations; in this sense, the tearing effect seems to enrich the eye area. We have not found any previous research in which this technique has been applied to the study of tears, so we are unable to compare our results. However, it is worth highlighting the work of Balsters et al. (2013), in which tears were presented as visual cues at a preattentive level and were still capable of arousing greater kindness, feelings of empathy, and connectedness. Our results are in line with these studies, as all of them point to tears functioning as a powerful visual cue that acts as a gaze magnet.

Another of our aims was to explore the relation between the cognitive empathy of the observers and the processing of tears in the calm crying faces. Interestingly, we found that a higher RMET score was significantly correlated with higher intensity of emotion only when visible tears were present, while the relationship was reversed in the absence of emotional crying. There is empirical evidence (Carr and Lutjemeier, 2005; Gery et al., 2009) that empathy is related to the ability to recognize emotions in emotional expressions, and such accurate emotional inference can be achieved during very short exposure to a facial expression. Accordingly, we found that a high level of cognitive empathy qualified people to discern and adequately label the non-crying face as being less intense and the crying face as transmitting higher emotional intensity. Interestingly, our results concerning empathy are in line with those of Harrison et al. (2007), who showed that sensitivity to the influence of pupil size, an autonomous signal related to tears in sad faces, correlated positively with the empathy score of the sample. In addition – and relevant for future research with broader samples in order to assure power – it would be interesting to examine the relation and causal direction among perceived emotional intensity, presence of tears, and cognitive empathy by means of structural equation modeling (Wang and Wang, 2020). In a recent mediation analysis (Küster, 2018), it was shown that visible tears produce an all-or-nothing effect where the intensity of crying does not appear to be a significant variable. Indeed, in the present study, we have found that the presence of a minimal signal of weeping was sufficient to provoke a measurable reaction in the observer.

Our observations regarding vulnerability to personality disorders and processing of tearful faces should be interpreted cautiously and received as suggestions to be put to the test in future studies with broader non-clinical samples and clinical populations. That said, it is noteworthy that significant correlations were detected only when the participants were judging tearful faces and that most are in line with data in the literature (clinical or otherwise). For instance, we found a positive association between higher paranoid ideation scores and the emotional intensity perceived in our calm crying faces; this is in accordance with a previously reported bias toward the perception of negative emotions in cases of clinical paranoia, with negatively biased interpretations of emotional ambiguity (Savulich et al., 2015). Regarding narcissism, which was correlated negatively with the “tearing effect,” we have stated in *Introduction* that individuals with narcissistic personality disorder (NPD) cry more than others. It is perhaps plausible that a person with a higher NPD trait score will interpret tears as more “normal” and less important, given that she/he is more accustomed to crying. Our results concerning the influence of vulnerability to personality disorders on the perceived sincerity of crying faces were even more relevant; on most of the scales, higher scores were associated with lower levels of sincerity attributed to the crying model. This was especially clear in the case of the personalities grouped in clusters A (strange and extravagant) and B (immature and emotionally unstable), thus showing that these personalities interpreted the crying behavior in a slightly different way. Lastly, the isolated positive correlation between a higher score for antisocial personality and the duration of fixations on the entire tearful face (global stimulus) is of special interest. We wonder whether this kind of personality increases the visual attention given to the whole face as a way of avoiding tearful eyes. This would support the recent observation that a higher psychopathy level is a significant predictor of reduced eye contact measured with eye tracking (Gehrer et al., 2020).

## Strengths and Limitations

This is the first study of an experimental line that employs an objective eye tracking protocol to evaluate emotional crying perception, and its results extend the existing behavioral data by introducing some physiological variables. To date, only one (recent) report has provided objective evidence of the tearing effect using psychophysiological measurements (Krivan et al., 2020). In our view, the present study represents a first step toward understanding crying as a visual signal of communication by means of the technology that best captures the particularities of this very special stimulus and has important social connotations. As a next step, future research should combine the psychophysical visual data obtained via eye tracking with electroencephalogram (EEG) records (e.g., event-related fixations and postsaccadic event-related potentials). Along with more traditional assessment of the socioemotional effect of tears, such research could lead to new hypotheses and new advances.

Regarding the limitations of the present work, the present design could be improved by examining the results in an additional control condition including other visual stimuli depicted in the faces of models instead of tears (e.g., a freckle, a wart, or a mole under the eye) in order to study differential gaze behavior and thus add useful physiological data to the behavioral results of Provine (2014, 2017). Moreover, we advise prudence when interpreting the correlational results: although the sample size was appropriate for the experimental study – as confirmed by the power analysis – the exploration of how personality variables are associated with facial recognition in the presence of visible tears will require a larger sample to draw solid conclusions. In addition, this study was performed with a limited number of visual stimuli (i.e., faces). We could have increased the number of stimuli to be more in line with other studies, but the selection was made with the aim of replicating and extending previous findings (van de Ven et al., 2016; Vingerhoets et al., 2016; Picó et al., 2020). Moreover, as mentioned in “Materials and Method,” the subjects were selected based on their ecological validity, i.e., they were calm crying in a spontaneous way. Finally, it should be taken into account that, due to availability (high female bias), we carried out our experiments in a purely female population; therefore, until the results are replicated with male participants, our conclusions should be applied to the general population with caution. In this respect, it should be pointed out that, according to Mulac et al. (1986), female dyads make much greater visual contact during interactions than male counterparts. This trend has been observed in other cultures (Wada, 1990) and is consistent with evidence that women are more sensitive non-verbal communicators (Rosenthal and DePaulo, 1979; Rosenthal, 1979) and exhibit greater sensitivity to non-verbal cues (Keeley-Dyreson et al., 1991) than men. Therefore, we advise caution in generalizing our conclusions on eye tracking results with respect to both genders when studying non-verbal behavior.

## Conclusion

Visible tears proved to be magnets for gaze during a face-viewing task. When they were present, the inspection pattern changed qualitatively and quantitatively, with participants becoming fully focused on the tears. The mere presence of a single teardrop running down the cheek was associated with increased emotional inference and a greater perception of sincerity. Interestingly, visible tears generated different reactions depending on the observer’s personality traits, with a positive relationship observed between cognitive empathy and the perception of greater emotional intensity in tearful faces. All in all, eye tracking technology seems to be an effective tool for studying the visual aspect of emotional crying, and we hope that the present study will be the first of many empirical works that investigate the interpersonal effects of tears. Additionally, we have commented on several of the methodological aspects that should be taken into account when using eye tracking technology to study non-verbal behavior, some of which have been neglected until now. Further exploration of the relationship between empathy and tear perception using eye tracking could be a fruitful avenue for future research.

## Data Availability Statement

The datasets generated for this study are available on request to the corresponding author.

## Ethics Statement

The studies involving human participants were reviewed and approved by the Comité de Ética de la Universidad de Valencia. The participants provided their written informed consent to participate in this study. Written informed consent was obtained from the individual(s) for the publication of any potentially identifiable images or data included in this article.

## Author Contributions

AP and MG conceived the study and wrote the manuscript. AP carried out the study and made the statistical analyses of the data. RE contributed to the interpretation of the study and made a critical revision of the draft. All authors contributed to the article and approved the submitted version.

## Conflict of Interest

The authors declare that the research was conducted in the absence of any commercial or financial relationships that could be construed as a potential conflict of interest.
